# Efficacy comparison of seven non-invasive brain stimulation techniques for upper limb motor dysfunction after stroke: a Bayesian network meta-analysis and systematic review

**DOI:** 10.3389/fneur.2025.1693537

**Published:** 2025-12-19

**Authors:** Yin Zheng, Jiazhen Liu, Xuxin Zhang, Jiani Xia, Bo Liu, Dapeng Li, Xuejiao Teng, Liye Lin, Kewei Jia, Yining Xu, Sitong Wu, Hong Ji

**Affiliations:** 1 Liaoning University of Traditional Chinese Medicine, Shenyang, Liaoning, China; 2 Affiliated Hospital of Liaoning University of Traditional Chinese Medicine, Shenyang, Liaoning, China; 3 School of Basic Medical Sciences, Liaoning University of Traditional Chinese Medicine, Shenyang, Liaoning, China; 4 Shengjing Hospital Affiliated to China Medical University, Shenyang, Liaoning, China

**Keywords:** Bayesian model, network meta-analysis, theta burst stimulation, transcranial direct current stimulation, transcranial magnetic stimulation

## Abstract

**Background:**

Stroke frequently causes upper limb dysfunction, impairing daily activities and quality of life. This study evaluates seven repetitive transcranial magnetic stimulation and transcranial direct current stimulation protocols for improving upper limb motor function, muscle tone, and daily living in stroke patients, providing evidence for optimizing non-invasive brain stimulation therapy.

**Methods:**

Computerized searches were conducted in the VIP database (VIP), Wan-fang database, China National Knowledge Infrastructure (CNKI), PubMed, SinoMed Database (CBM), Cochrane Library, and Web of Science databases to identify publicly published randomized controlled trials on different non-invasive brain stimulation techniques for upper limb motor dysfunction after stroke. The search period was up to November 2024. The Cochrane Risk of Bias tool (version 5.4.0) was used to assess the quality of the included studies. R software (version 4.1.1) was used to perform Bayesian network meta-analysis for data comparison and ranking.

**Results:**

A total of 28 studies were included, with a total sample size of 1,340 patients, encompassing 7 non-invasive brain stimulation techniques. Probability ranking results indicated the following: for Fugl-Meyer Assessment for Upper Extremity (FMA-UE), the top three rankings were high-frequency repetitive transcranial magnetic stimulation (HF-rTMS) (29%), continuous theta burst stimulation (cTBS) (27%), and anodal transcranial direct current stimulation (aDCS) (17.5%); for Barthel Index (BI), the top three were aDCS (71.5%), low-frequency repetitive transcranial magnetic stimulation(LF-rTMS) (20.9%), and HF-rTMS (4.4%); for Wolf Motor Function Test (WMFT), the top three were aDCS (63.6%), cTBS (13%), and HF-rTMS (9.1%); for Modified Ashworth Scale (MAS), the top three were intermittent theta burst stimulation (iTBS) (42%), LF-rTMS (24%), and cTBS (16%); for Action Research Arm Test (ARAT), the top three were iTBS (72.6%), aDCS (22.3%), and LF-rTMS (2.8%).

**Conclusion:**

Based on the network meta-analysis results and probability ranking evidence, HF-rTMS is most likely to be the most effective intervention for restoring motor function (FMA-UE); aDCS may rank first for both activities of daily living (BI) and motor task performance (WMFT); iTBS appears beneficial for improving muscle tone regulation (MAS) and fine motor ability (ARAT). However, the results for ARAT (6 studies) and MAS (9 studies), based on a smaller number of studies, should be interpreted with caution due to limited evidence.

**Systematic review registration:**

https://www.crd.york.ac.uk/PROSPERO/, identifier CRD420251019764.

## 1 Introduction

Stroke, also known as cerebrovascular accident, is a cerebrovascular disease caused by various pathological mechanisms such as cerebrovascular occlusion or rupture, leading to focal or global neurological impairment. According to World Health Organization (WHO) reports, stroke causes approximately 5.5 million deaths globally each year, and China sees about 2 million new stroke cases annually (1). Among these, 55–75% of individuals have upper limb motor dysfunction (2, 3). Clinical manifestations primarily include muscle weakness, abnormally increased muscle tone, restricted joint range of motion, and altered corticospinal tract excitability. These pathological changes often lead to reduced ability in activities of daily living, significantly diminishing patients’ quality of life.

Although physical therapy modalities, represented by task-oriented training, are widely adopted in clinical practice, their efficacy remains limited by factors such as long treatment cycles and dependence on patient compliance. For intractable dysfunction, invasive interventions like deep brain stimulation (DBS) can improve motor function, but issues such as the risk of postoperative infection and the cost-effectiveness of unilateral surgery make it difficult to become a universal option. Over the past two decades, non-invasive brain stimulation (NIBS) techniques have gradually evolved into key intervention strategies for improving post-stroke motor function. This technology, based on the theory of interhemispheric inhibition competition, modulates excitability levels in bilateral cerebral hemispheres through electrical or magnetic energy (4, 5). Currently, NIBS protocols commonly used in clinical practice mainly include repetitive transcranial magnetic stimulation (rTMS) and transcranial direct current stimulation (tDCS).

Repetitive transcranial magnetic stimulation (rTMS), as a non-invasive and safe brain stimulation technique, has been widely applied in the field of upper limb motor function rehabilitation after stroke (6). Its treatment protocols are mainly categorized into four types based on stimulation frequency: high-frequency rTMS (>1 Hz), low-frequency rTMS (≤1 Hz), intermittent theta burst stimulation (iTBS), and continuous theta burst stimulation (cTBS). Transcranial direct current stimulation (tDCS) modulates cortical excitability by applying a low-intensity direct current, and its mechanism of action may involve polarization changes in neuronal membrane potentials (7). Common clinical protocols include anodal excitatory stimulation, bipolar balanced stimulation, and cathodal inhibitory stimulation.

Network meta-analysis systematically integrates evidence from studies meeting inclusion criteria, enables multi-dimensional comparisons of interventions with common characteristics, and ranks interventions based on the weight of outcome indicators to determine the optimal treatment regimen, thereby providing evidence-based support for clinical decision-making. This study intends to use this method to systematically evaluate the effects of seven intervention protocols of rTMS and tDCS on upper limb motor function, muscle tone, and activities of daily living in stroke patients, aiming to provide a scientific basis for the clinical selection of NIBS techniques.

## 2 Materials and methods

### 2.1 Data sources

Computerized searches were conducted in the VIP, Wanfang, CNKI, PubMed, CBM, Cochrane Library, and Web of Science databases to identify literature relevant to this study. The search period was up to November 2024. The main search terms included Chinese and English terms such as stroke, cerebrovascular accident, hemorrhagic stroke, cerebral infarction, apoplexy, transcranial magnetic stimulation, transcranial direct current stimulation, theta burst stimulation, upper limb motor dysfunction, etc. The search was performed by combining subject headings, keywords, and abstracts, as detailed in Table 1.

**Table 1:** PubMed search strategy.

Databases	PubMed	Search fields
Search #	Search query	
#1	(“Stroke”[Mesh] OR “Cerebrovascular Accident”[tiab] OR “Hemorrhagic Stroke”[tiab] OR “Ischemic Stroke”[tiab] OR “Brain Infarction”[Mesh] OR “Cerebral Infarction”[tiab] OR “Post-stroke”[tiab])	All fields
#2	(“Upper Extremity”[Mesh] OR “Arm”[tiab] OR “Hand”[tiab] OR “Upper Limb”[tiab]) AND (“Motor Disorders”[Mesh] OR “Motor Dysfunction”[tiab] OR “Motor Recovery”[tiab] OR “Fugl-Meyer Assessment”[tiab])	All fields
#3	(“Transcranial Magnetic Stimulation”[Mesh] OR “TMS”[tiab] OR “Repetitive Transcranial Magnetic Stimulation”[tiab] OR “rTMS”[tiab])	All fields
#4	(“Transcranial Direct Current Stimulation”[Mesh] OR “tDCS”[tiab] OR “Anodal tDCS”[tiab] OR “Cathodal tDCS”[tiab])	All fields
#5	(“Theta Burst Stimulation”[Mesh] OR “TBS”[tiab] OR “Continuous Theta Burst Stimulation”[tiab] OR “cTBS”[tiab] OR “Intermittent Theta Burst Stimulation”[tiab] OR “iTBS”[tiab])	All fields
#6	(“Transcranial Alternating Current Stimulation”[tiab] OR “tACS”[tiab] OR “Transcranial Random Noise Stimulation”[tiab] OR “tRNS”[tiab])	All fields
#7	(“Transcranial Pulsed Current Stimulation”[tiab] OR “tPCS”[tiab] OR “Pulsed Transcranial Stimulation”[tiab])	All fields
#8	(“Transcranial Focused Ultrasound Stimulation”[tiab] OR “TFUS”[tiab] OR “Low-Intensity Focused Ultrasound”[tiab] OR “LIFUP”[tiab])	All fields
#9	(“Non-Invasive Brain Stimulation”[Mesh] OR “NIBS”[tiab] OR “Neuromodulation Techniques”[tiab])	All fields
#10	#3 OR #4 OR #5 OR #6 OR #7 OR #8 OR #9	All fields
#11	(“Randomized Controlled Trial”[pt] OR “Controlled Clinical Trial”[pt] OR “Randomized”[tiab] OR “Randomly”[tiab] OR “Placebo”[tiab] OR “Clinical Trial”[pt]) NOT (“Animals”[Mesh] NOT “Humans”[Mesh])	All fields
#12	#1 AND #2 AND #10 AND #11	All fields

### 2.2 Inclusion criteria

#### 2.2.1 Study type

Randomized Controlled Trials (RCTs). Languages were restricted to Chinese and English. Blinding was not restricted.

#### 2.2.2 Study population

Stroke patients with upper limb dysfunction diagnosed according to the Chinese Diagnostic Criteria for Major Cerebrovascular Diseases (2019) (8) or the Diagnostic Criteria of the Fourth National Cerebrovascular Conference (1995) (9), or meeting WHO stroke diagnostic criteria.

#### 2.2.3 Interventions

Comparisons between rTMS/tDCS and a sham group, comparisons with conventional therapy, or direct comparisons between rTMS and tDCS.

#### 2.2.4 Outcome indicators

① Fugl-Meyer Assessment for Upper Extremity (FMA-UE): This scale serves as a standardized assessment tool for upper limb motor function after stroke, with a total score set at 66 points. Its score is positively correlated with the level of upper limb motor function recovery, meaning a higher score indicates better functional recovery. ② Wolf Motor Function Test (WMFT): By combining timed tasks and functional tasks, the WMFT can quantitatively analyze upper limb motor ability, covering joint-specific movements and overall limb coordination assessment. This tool is often used in clinical research involving stroke patients or those with significant upper limb impairment. Its score is generally positively correlated with the degree of motor function improvement. ③ Action Research Arm Test (ARAT): The ARAT includes four dimensions: grasp (6 items), grip (4 items), pinch (6 items), and gross movement (3 items). Each item is scored on a 4-point scale (0–3), where 0 represents inability to perform the action and 3 indicates normal performance. The total score is 57 points, with higher scores indicating better upper limb function recovery. ④ Modified Ashworth Scale (MAS): The MAS uses a 6-point scoring system (0, 1, 1+, 2, 3, 4) to assess the degree of abnormal upper limb muscle tone. An increase in grade is significantly associated with pathological enhancement of muscle tone. ⑤ Barthel Index (BI): As a core assessment tool for activities of daily living, the BI has a maximum score of 100 points. Its score generally reflects the recovery level of the patient’s independent living ability, with higher scores indicating daily living function closer to normal.

### 2.3 Exclusion criteria

① Non-Chinese or non-English literature; ② Intervention duration <2 weeks; ③ Experience summaries, review articles; ④ Animal experimental studies; ⑤ Purely exploratory literature; ⑥ Duplicate publications; ⑦ Literature for which the full text is unavailable; ⑧ Studies with incomplete trial data where the mean and standard deviation of outcome measures cannot be obtained.

### 2.4 Literature screening

EndNote software was first used to screen the imported records for duplicates. Search results from different databases were integrated, and full texts were downloaded to remove literature that did not meet the inclusion criteria during re-screening. If key data were missing from the literature, contact with the original authors was attempted. The above work, including data extraction using a pre-designed form, was independently performed by two researchers. Any disagreements were resolved through consultation with a third researcher.

### 2.5 Data extraction

Extracted content included: author, publication year, country, sex, sample size, age, interventions, course of treatment, and outcome measures. To ensure that all included studies reported on independent patient cohorts, a strict and systematic check was performed. Although some studies originated from the same institution, their patient recruitment periods did not overlap, patients were from hospitals in different regions, and there were identifiable differences in baseline characteristics. Therefore, we are confident that the currently included analysis data do not have issues of population duplication. The sources of patients from the same institution are detailed in Supplementary Table 1.

### 2.6 Quality assessment

The methodological quality of the included studies was assessed using the risk of bias tool recommended by the Cochrane Handbook for Systematic Reviews of Interventions (Version 5.4.0). The assessment items included: random sequence generation, allocation concealment, blinding, incomplete outcome data, selective reporting, and other potential sources of bias. Each item was judged as high risk, unclear risk, or low risk.

### 2.7 Statistical analysis

Review Manager (RevMan) 5.4 software was used for the risk of bias assessment. Network meta-analysis was performed using R software to compare the efficacy of the seven intervention protocols directly and indirectly. For continuous data, the mean difference (MD) and its 95% credible interval (CrI) were used. Heterogeneity was assessed using the *I*^2^ statistic; an *I*^2^ ≥ 50% indicated significant heterogeneity among studies, necessitating further subgroup analysis and sensitivity analysis to identify the source of heterogeneity. If the source could not be determined, a random-effects model was employed from both methodological and statistical perspectives. A Bayesian model was established using R. The model was fitted using four Markov chains, with an initial value of 2.5 and the number of iterations set to 20,000. The potential scale reduction factor (PSRF) was calculated; a value approaching 1.0 indicated satisfactory convergence; otherwise, iterations were continued until model convergence was achieved. The choice between random-effects and fixed-effects models was based on the deviance information criterion (DIC). The DIC values for both random-effects and fixed-effects models were calculated, and generally, the model with the smaller DIC value was selected as the optimal model. If closed loops existed in the network graph, the inconsistency between direct and indirect evidence was assessed. Rankograms were plotted; a higher probability of being Rank 1 indicated that the intervention was more likely to be the best.

## 3 Results

### 3.1 Literature search results

**Figure 1:**
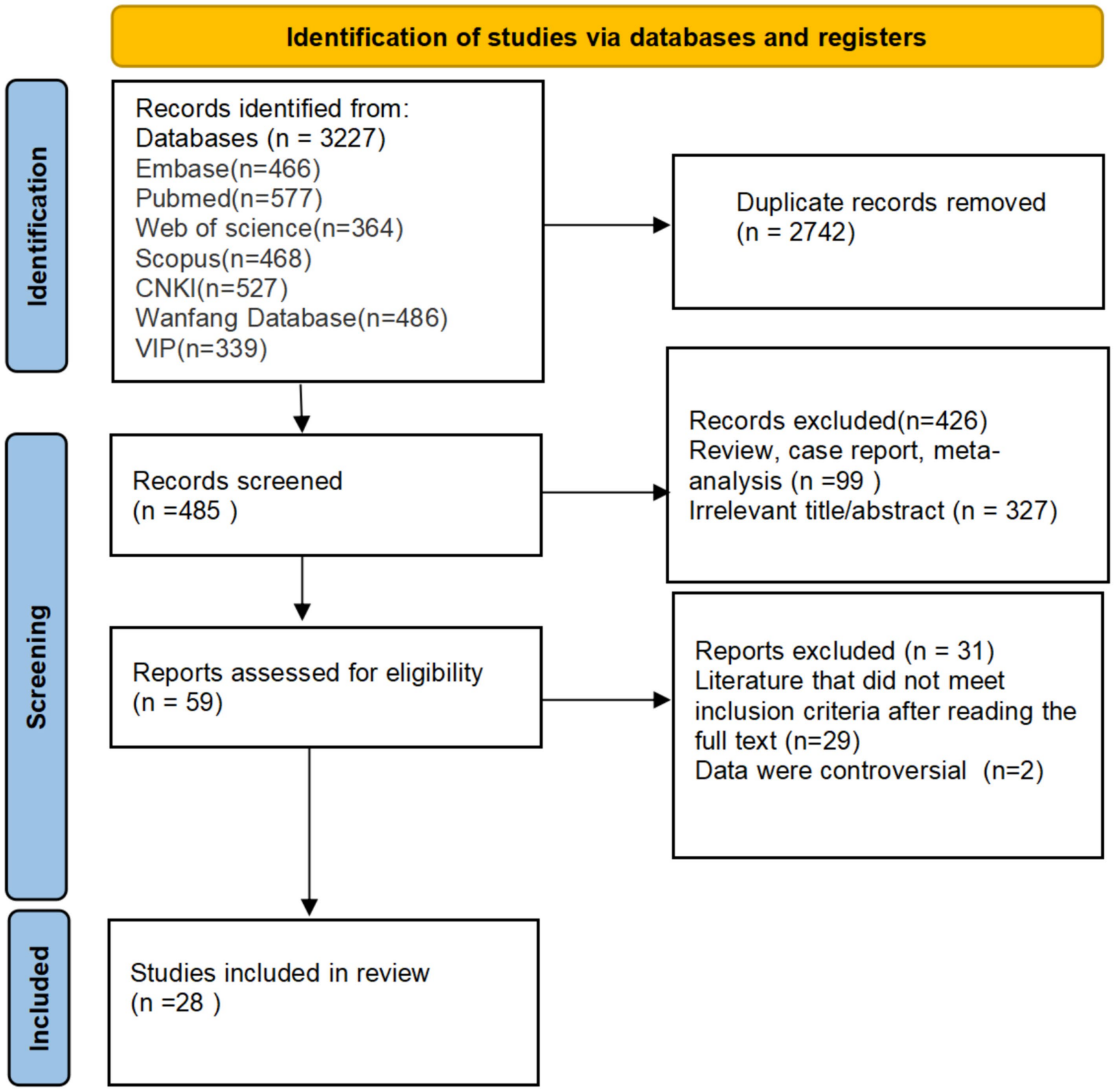
Flowchart of study selection for inclusion.

The initial search yielded 3,227 articles. After deduplication and exclusion of non-eligible studies using EndNote software, 3,199 articles were removed. Ultimately, 28 articles were included (10–37), comprising 15 English and 13 Chinese publications. The literature screening flow is shown in Figure 1.

### 3.2 Basic characteristics of included studies and risk of bias assessment results

**Figure 2:**
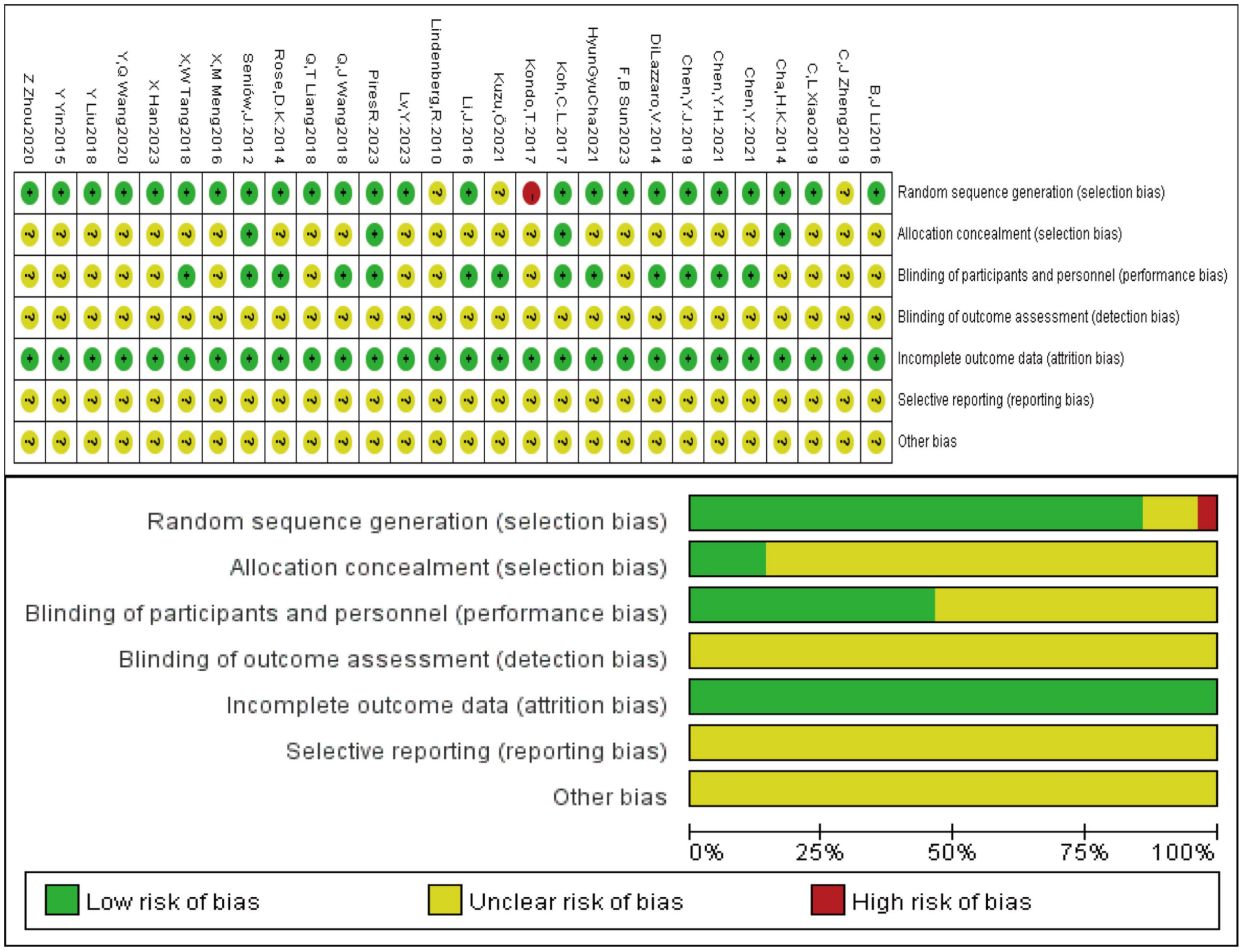
Risk of bias assessment of the included studies.

A total of 28 studies with a sample size of 1,340 patients were included. Twenty-six studies reported FMA (10, 11, 13–20, 22–37), 10 studies reported WMFT (14–16, 19, 20, 24, 26–28, 37), 6 studies reported ARAT (12, 13, 18, 20, 22, 23), 9 studies reported MAS (13, 18, 21–23, 25, 31, 32, 35, 36), and 12 studies reported BI (13, 15, 18, 19, 21, 29–31, 33–36). Twenty-four studies described specific randomization methods such as random number tables or computer randomization (10–13, 15–18, 20–23, 26–37). Four studies used sealed envelope allocation (10, 11, 13, 28), and 13 studies implemented blinding (10, 12, 13, 17, 20–23, 25–28, 33). The basic characteristics of the included studies are shown in Table 2. The rTMS parameters and treatment protocols are detailed in Table 3, and the tDCS study characteristics are shown in Table 4. The risk of bias assessment is presented in Figure 2.

**Table 2:** Basic characteristics of the included studies.

Author (year)	Country	*N* (M/F)		Average age/year		Intervention		Treatments	Outcome
		T	C	T	C	T	C		
Pires R. (2023) (10)^a^	BRA	(8, 12)/(10, 10)/(6, 11)		58.25 ± 8.74 59.42 ± 9.96 56.81 ± 10.18		aDCS, cDCS, placebo		2 weeks	①
Cha H. K. (2014) (11)	KOR	–		59.8 ± 11.4	57.8 ± 9.9	B-tDCS	Conventional therapy	4 weeks	①
DiLazzaro V. (2014) (12)	ITA	(5, 2)/(4, 3)		66.43 ± 5.96	71.71 ± 5.25	B-tDCS	Placebo	2 weeks	①③④⑤
Koh C. L. (2017) (13)	CHN	(8, 6)/(7, 4)		55.3 ± 11.5	56.9 ± 13.5	B-tDCS	Placebo	6 weeks	①③④⑤
Lindenberg R. (2010) (14)	USA	(8, 2)/(7, 3)		61.7 ± 14.7	55.8 ± 12.9	B-tDCS	Placebo	2 weeks	①②
X Han (2023) (15)	CHN	(34, 13)/(34, 15)		67.51 ± 10.72	67.04 ± 10.73	aDCS	Placebo	4 weeks	①②⑤
F. B Sun (2023) (16)	CHN	(13, 7)/(11, 9)		59.90 ± 10.80	61.35 ± 9.82	aDCS	Placebo	6 weeks	①②
Q. J. Wang (2018) (17)	CHN	(36, 7)/(32, 8)		62.1 ± 5.8	64.2 ± 4.9	aDCS	Placebo	8 weeks	①
Y. Yin (2015) (18)	CHN	(27, 13)/(30, 10)		55.70 ± 12.32	57.68 ± 13.54	aDCS	Placebo	4 weeks	①③④⑤
C. J. Zheng (2019) (19)	CHN	(27, 22)/(28, 19)		59.8 ± 8.3	61.1 ± 7.4	aDCS	Placebo	4 weeks	①②⑤
Rose D. K. (2014) (20)	CHN	9/10		64.7 ± 7.0	64.6 ± 9.0	LF-rTMS	Placebo	4 weeks	①②③
Chen Y. (2021) (21)	CHN	(13, 3)/(12, 4)		57.38 ± 8.04	51.44 ± 9.19	iTBS	Placebo	2 weeks	④⑤
Chen Y. H. (2021) (22)	CHN	(8, 4)/(10, 1)		54.36 ± 10.56	48.95 ± 9.63	iTBS	Placebo	3 weeks	①③④
Chen Y. J. (2019) (23)	CHN	(7, 4)/(7, 4)		52.9 ± 11.1	52.6 ± 8.3	iTBS	Placebo	2 weeks	①③④
Kondo T. (2017) (24)	JPN	(51, 20)/(20, 20)		62.3 ± 12.5	60.0 ± 14.2	LF-rTMS	cTBS	2 weeks	①②
Kuzu Ö. 2021 (25)^a^	TUR	(6, 1)/(4, 3)/(2, 4)		61.3 ± 9.8 56.3 ± 11.5 65 ± 4.6		cTBS, LF-rTMS, placebo		2 weeks	①④
Li J. (2016) (26)^a^	CHN	(30, 12)/(29, 14)/(28, 14)		57.87 ± 12.89 54 ± 13.35 53.13 ± 13.27		LF-rTMS, HF-rTMS, placebo		2 weeks	①②
HyunGyuCha (2021) (27)	KOR	(13, 7)/(12, 8)		67.64 ± 7.16	69.09 ± 6.04	LF-rTMS	HF-rTMS	2 weeks	①②
Seniów J. (2012) (28)	POL	(12, 8)/(14, 6)		63.5 ± 8.9	63.4 ± 9.2	LF-rTMS	Placebo	3 weeks	①②
B. J. Li (2016) (29)	CHN	(12, 8)/(11, 9)		51.52 ± 7.08	51.42 ± 6.56	LF-rTMS	Conventional therapy	2 weeks	①⑤
Q. T. Liang (2018) (30)	CHN	(25, 14)/(22, 17)		64.3 ± 11.8	65.5 ± 10.5	HF-rTMS	Conventional therapy	8 weeks	①⑤
Y. Liu (2018) (31)	CHN	(5, 5)/(9, 4)		56.90 ± 9.02	55.38 ± 8.40	LF-rTMS	Conventional therapy	6 weeks	①④⑤
X. M. Meng (2016) (32)^a^	CHN	(11, 3)/(12, 5)/(10, 4)		57.43 ± 13.26 55.12 ± 12.88 51.21 ± 14.13		LF-rTMS, HF-rTMS, placebo		2 weeks	①④
X. W Tang (2018) (33)	CHN	(7, 1)/(7, 1)		53.75 ± 10.77	55.62 ± 14.55	iTBS	Placebo	2 weeks	①⑤
Y. Q. Wang (2020) (34)	CHN	(20, 16)/(21, 15)		51.98 ± 6.94	53.09 ± 6.83	LF-rTMS	Placebo	4 weeks	①⑤
C. L. Xiao (2019) (35)	CHN	(13, 7)/(12, 7)		60.4 ± 11.77	57.68 ± 10.66	HF-rTMS	Placebo	2 weeks	①④⑤
Z. Zhou (2020) (36)	CHN	(22, 8)/(19, 9)		59.73 ± 10.41	61.75 ± 11.43	HF-rTMS	Placebo	3 weeks	①④⑤
Lv Y. (2023) (37)	CHN	(11, 7)/(10, 8)		57.3 ± 11.5	57.8 ± 17.6	LF-rTMS	Conventional therapy	4 weeks	①②

“–” Indicates that the intervention was not applied or reported for the specific outcome metric. N is the sample size; M is Male; F is Female; T is the experimental group; C is the control group. The superscript “a” indicates a three-arm trial. aDCS: anodal transcranial direct current stimulation; cDCS: cathodal transcranial direct current stimulation; Bipolar DCS: bipolar transcranial direct current stimulation; Conventional therapy (medication and rehabilitation therapy); LF-rTMS: low-frequency repetitive transcranial magnetic stimulation; iTBS: intermittent theta burst stimulation; cTBS: continuous theta burst stimulation; HF-rTMS: high-frequency repetitive transcranial magnetic stimulation. ① Fugl-Meyer Assessment for Upper Extremity (FMA-UE); ② Wolf Motor Function Test (WMFT); ③ Action Research Arm Test (ARAT); ④ Modified Ashworth Scale (MAS); ⑤ Barthel Index (BI).

**Table 3:** rTMS parameters and treatment protocols in the included literature.

Author(year)	Stimulation frequency (Hz)	Stimulation intensity	Total pulses	Stimulation site	Coil type	Treatment protocol and duration
Rose D. K. (2014) (20)	1	100%rMT	1,200	Contralesional M1	Figure-of-8 coil	20 min/session, 4 sessions/week for 4 weeks
Chen Y. (2021) (21)	50–5	80%AMT	600	Ipsilesional Cerebellum	Figure-of-8 coil	200 s/session, 5 sessions/week for 2 weeks
Chen Y. H. (2021) (22)	50–5	80%AMT	1,200	Contralesional M1	Figure-of-8 coil	200 s/session, 5 sessions/week for 3 weeks
Chen Y. J. (2019) (23)	50–5	80%AMT	600	Ipsilesional M1	Figure-of-8 coil	200 s/session, 5 sessions/week for 2 weeks
Kondo T. (2017) (24)	1/50–5	90%RMT/80%AMT	2,400	Motor cortex	Figure-of-8 coil	20 min/session (1 Hz), 6 sessions/week for 2 weeks; 160 s/session (iTBS), 6 sessions/week for 2 weeks
Kuzu Ö. 2021 (25)^a^	1/50–5	90%RMT/80%AMT	1200/600	Contralesional M1	Figure-of-8 coil	200 s/session, 5 sessions/week for 2 weeks
Li J. (2016) (26)^a^	1/10	80%AMT	1000/1350	Contralesional M1/Ipsilesional M1	Circular coil	20 min/session, 5 sessions/week for 2 weeks
HyunGyuCha (2021) (27)	1/10	90%RMT	1000/1350	Contralesional M1/Ipsilesional M1	Figure-of-8 coil	20 min/session, 5 sessions/week for 2 weeks
Seniów J. (2012) (28)	1	90%RMT	1,200	Contralesional M1	Figure-of-8 coil	30 min/session, 5 sessions/week for 3 weeks
B. J. Li (2016) (29)	1	80%AMT	600	Contralesional M1	Figure-of-8 coil	20 min/session, 5 sessions/week for 2 weeks
Q. T Liang (2018) (30)	3	90% ~ 120%AMT	900	Ipsilesional C3, C4	Figure-of-8 coil	20 min/session, continued for 2 weeks followed by 2 days rest, total 8 weeks
Y. Liu (2018) (31)	1	90%AMT	1,200	Contralesional M1	Figure-of-8 coil	20 min/session, 5 sessions/week for 6 weeks
X. M. Meng (2016) (24)^a^	1/10	80%AMT	1000/1350	Contralesional M1/Ipsilesional M1	Circular coil	20 min/session, 5 sessions/week for 2 weeks
X. W. Tang (2018) (33)	50–5	70%RMT	600	Ipsilesional M1	Circular coil	200 s/session, 5 sessions/week for 2 weeks
Y. Q. Wang (2020) (34)	1	80%AMT	600	Contralesional M1	Figure-of-8 coil	20 min/session, 5 sessions/week for 4 weeks
C. L. Xiao (2019) (35)	10	90%RMT	900	Ipsilesional C3, C4	Figure-of-8 coil	20 min/session, 5 sessions/week for 2 weeks
Z. Zhou (2020) (36)	5	80%RMT	1,200	Ipsilesional M1	Figure-of-8 coil	5 min/session, 5 sessions/week for 3 weeks
Lv Y. (2023) (37)	1	80%RMT	1,200	Contralesional M1	Figure-of-8 coil	20 min/session, 5 sessions/week for 4 weeks

RMT = Resting Motor Threshold; AMT = Active Motor Threshold; M1 = Primary Motor Cortex; C3, C4: International 10–20 system for EEG electrode placement, 2 cm posterior to the M1 point. The superscript “a” indicates a three-arm trial.

**Table 4:** Characteristics of tDCS studies in the included literature.

Author (year)	T	C	Treatment course (Total sessions/Duration)
Pires R. (2023) (10)^a^	Anode placed over the ipsilesional motor cortex (C3 or C4 site), cathode placed over the contralateral supraorbital area./Cathode placed over the contralesional motor cortex (C3 or C4 site), anode placed over the ipsilesional supraorbital area./Sham group: Electrode placement identical to the anodal tDCS group.		10 sessions/2 weeks
Cha H. K. (2014) (11)	Anode placed over the ipsilesional motor cortex (C3 or C4 site), cathode placed over the contralateral supraorbital area.	Conventional therapy	20 sessions/4 weeks
DiLazzaro V. (2014) (12)	Anode placed over the ipsilesional motor cortex (C3 or C4 site), cathode placed over the contralesional motor cortex (C3 or C4 site).	Placebo	10 sessions/2 weeks
Koh C. L. (2017) (13)	Anode placed over the M1 area of the affected hemisphere (ipsilesional), cathode placed over the M1 area of the unaffected hemisphere (contralesional).	Placebo	24 sessions/6 weeks
Lindenberg R. (2010) (14)	Anode placed over the M1 area of the affected hemisphere (ipsilesional), cathode placed over the M1 area of the unaffected hemisphere (contralesional).	Placebo	10 sessions/2 weeks
X. Han (2023) (15)	Anode placed over the contralateral precentral gyrus (upper limb motor area), cathode placed over the ipsilateral shoulder.	Placebo	20 sessions/4 weeks
F. B. Sun (2023) (16)	Anode placed over the ipsilesional motor cortex (C3 or C4 site), cathode placed over the contralateral supraorbital area.	Placebo	20 sessions/6 weeks
Q. J. Wang (2018) (17)	Anode placed over the ipsilesional motor cortex (C3 or C4 site), cathode placed over the contralateral supraorbital area.	Placebo	20 sessions/6 weeks
Y. Yin (2015) (18)	Anode placed over the contralateral precentral gyrus (upper limb motor area), cathode placed over the ipsilateral shoulder.	Placebo	20 sessions/4 weeks
C. J. Zheng (2019) (19)	Anode placed over the M1 area of the affected hemisphere, cathode placed over the contralateral shoulder.	Placebo	20 sessions/4 weeks

T is the experimental group; C is the control group; M1 = Primary Motor Cortex; C3, C4: International 10–20 system for EEG electrode placement, 2 cm posterior to the M1 point. The superscript “a” indicates a three-arm trial.

### 3.3 Convergence evaluation and evidence network

**Figure 3:**
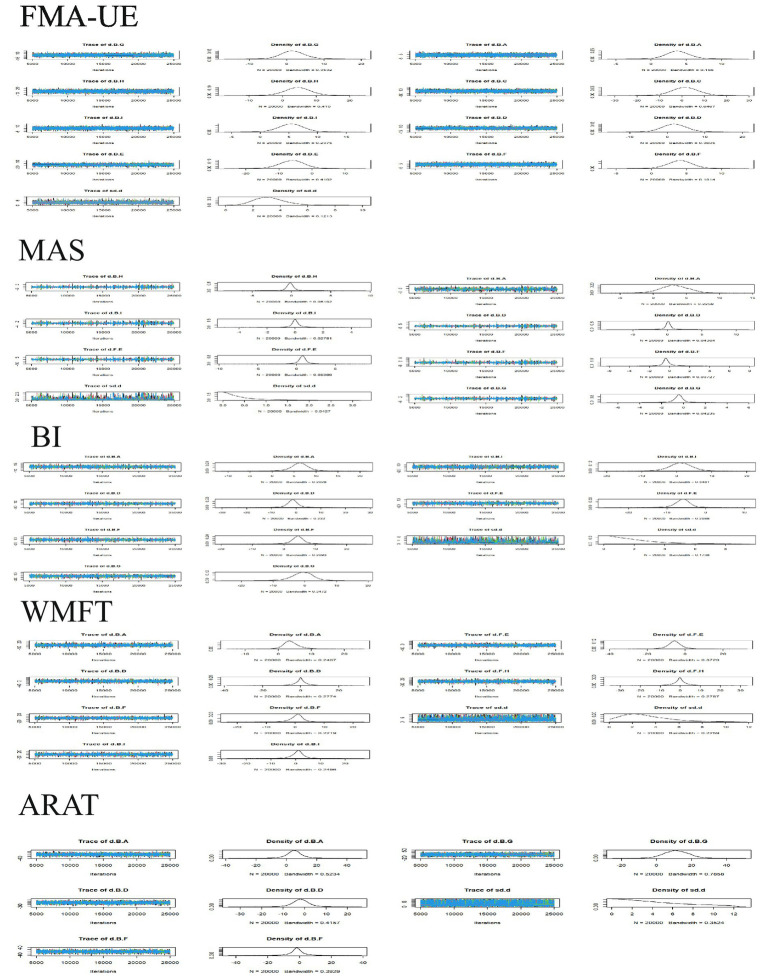
Trace plot and density plot for each outcome indicator. Trace Plots: The horizontal axis represents the number of iterations, and the vertical axis represents the parameter value range. The multi-colored curves represent the paths of independent Markov chains, reflecting parameter space exploration and convergence. Density Plots: The horizontal axis represents possible parameter values. The vertical axis represents probability density. The total area under the curve integrates to 1. A higher and narrower peak indicates a greater probability of the parameter values in that region.

**Figure 4:**
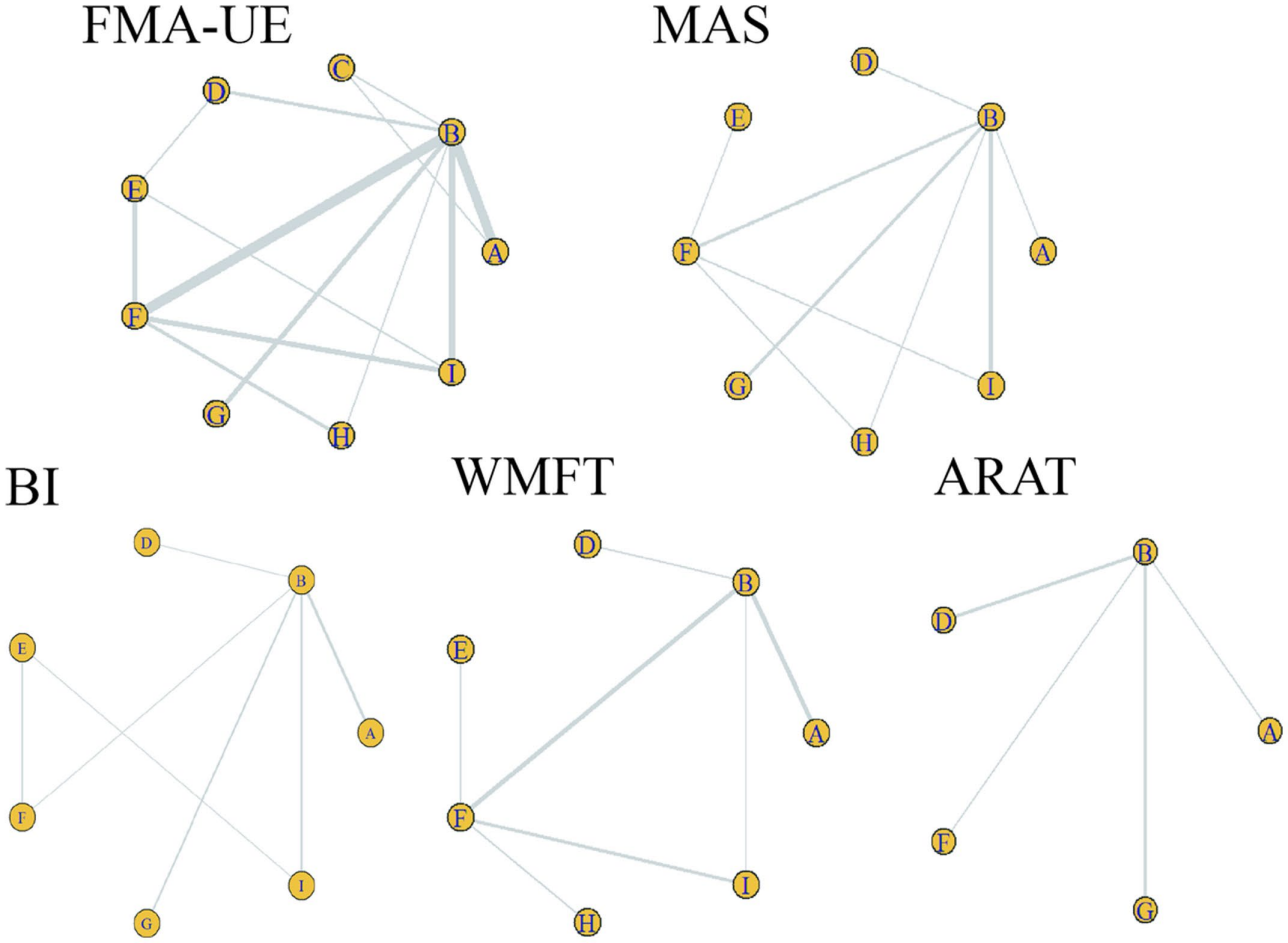
Evidence network for each outcome indicator. Circles represent different interventions; circle size represents sample size. Line thickness represents the number of studies comparing two interventions. A line connecting two points indicates a direct comparison relationship between those two interventions. A: anodal transcranial direct current stimulation (aDCS), B: Sham group, C: cathodal transcranial direct current stimulation (cDCS), D: bipolar transcranial direct current stimulation (bipolar DCS), E: Conventional therapy, F: low-frequency repetitive transcranial magnetic stimulation (LF-rTMS), G: intermittent theta burst stimulation (iTBS), H: continuous theta burst stimulation (cTBS), I: high-frequency repetitive transcranial magnetic stimulation (HF-rTMS). FMA-UE: Fugl-Meyer Assessment for Upper Extremity; WMFT: Wolf Motor Function Test; ARAT: Action Research Arm Test; MAS: Modified Ashworth Scale; BI: Barthel Index.

Consistency analysis for each outcome indicator showed that all PSFR values were less than 1.05, indicating good model convergence. Therefore, the network meta-analysis was performed under the consistency model. Specifically, the maximum number of iterations for the trace plots was 50,000. Most traces were stable, indicating good convergence of the Markov chains after multiple iterations, with estimates tending to stabilize and high reliability of the simulation results. Density plots were mostly unimodal; unimodality indicates a central tendency, with the peak corresponding to the most probable value. See Figure 3. The included studies encompassed the following treatments: A: anodal transcranial direct current stimulation (aDCS), B: Sham group, C: cathodal transcranial direct current stimulation (cDCS), D: bipolar transcranial direct current stimulation (bipolar DCS), E: Conventional therapy, F: low-frequency repetitive transcranial magnetic stimulation (LF-rTMS), G: intermittent theta burst stimulation (iTBS), H: continuous theta burst stimulation (cTBS), I: high-frequency repetitive transcranial magnetic stimulation (HF-rTMS). Sham stimulation in most studies was confirmed to effectively maintain participant blinding and met ethical and experimental safety requirements. Studies using sham stimulation were combined into a Sham group to facilitate comparison with active interventions. Specific protocols for sham stimulation in different studies are detailed in Supplementary Table 2. In Figure 4, line thickness represents the number of studies comparing two interventions. Lines indicate a direct comparison relationship between two interventions, and node size represents sample size.

### 3.4 Model selection for each outcome indicator

Table 5 summarizes the model fitting of the consistency model under fixed-effects and random-effects models for each outcome indicator, including the deviance information criterion (DIC), pD, Dbar, and *I*^2^. Based on the model goodness-of-fit and heterogeneity indicators, the optimal model was determined for each outcome for subsequent analysis.

**Table 5:** Goodness-of-fit and heterogeneity assessment results for the consistency models under different outcome indicators.

Outcome indicator	Model type	DIC	Dbar	pD	*I* ^2^	Model selection
FMA-UE	Random-effects	101.84282	57.16591	44.67691	4%	Random-effects
	Fixed-effect	112.26391	78.22162	34.04230	30%	
MAS	Random-effects	37.11654	19.66542	17.45112	3%	Fixed-effect
	Fixed-effect	35.30863	19.34743	15.9612	2%	
BI	Random-effects	41.99948	22.35112	19.64837	0%	Fixed-effect
	Fixed-effect	40.35334	22.33564	18.01770	0%	
WMFT	Random-effects	41.99307	22.19729	19.79578	10%	Random-effects
	Fixed-effect	44.70365	28.70218	16.00146	30%	
ARAT	Random-effects	21.48593	10.77087	10.71506	0%	Fixed-effect
	Fixed-effect	20.16966	10.16190	10.00776	0%	

DIC is the Deviance Information Criterion value; Dbar is the posterior mean deviance; pD is the effective number of parameters. FMA-UE: Fugl-Meyer Assessment for Upper Extremity; WMFT: Wolf Motor Function Test; ARAT: Action Research Arm Test; MAS: Modified Ashworth Scale; BI: Barthel Index.

### 3.5 Network meta-analysis results

**Figure 5:**
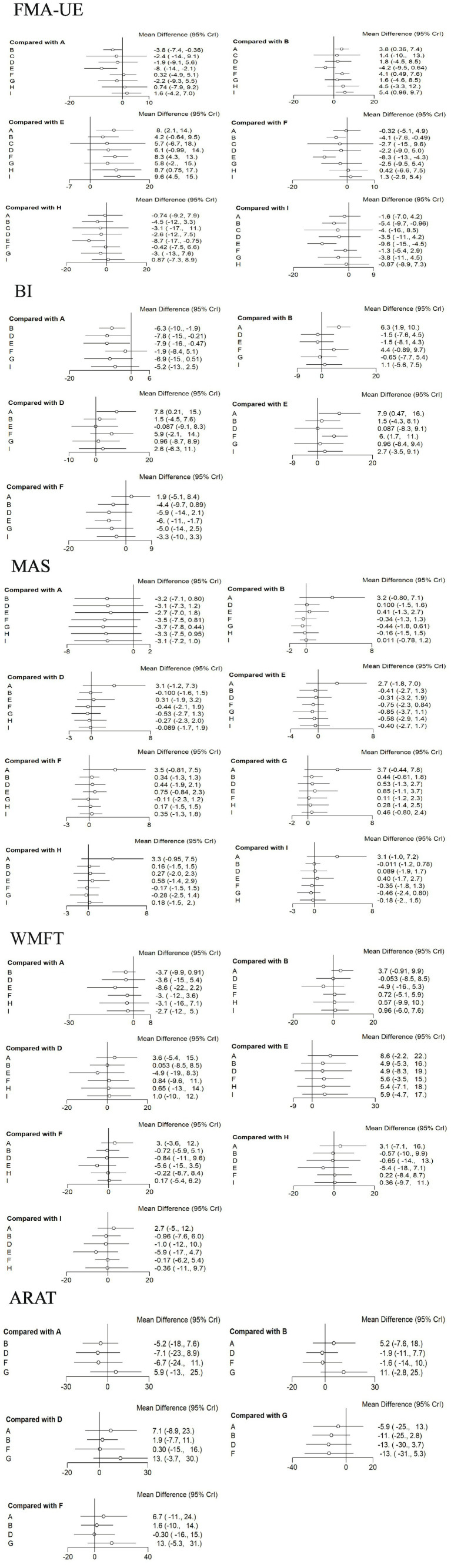
Forest plots for each outcome indicator. Each point represents the OR or MD of the intervention; the line through the point represents the 95% CrI. A: anodal transcranial direct current stimulation (aDCS), B: Sham group, C: cathodal transcranial direct current stimulation (cDCS), D: bipolar transcranial direct current stimulation (bipolar DCS), E: Conventional therapy, F: low-frequency repetitive transcranial magnetic stimulation (LF-rTMS), G: intermittent theta burst stimulation (iTBS), H: continuous theta burst stimulation (cTBS), I: high-frequency repetitive transcranial magnetic stimulation (HF-rTMS). FMA-UE: Fugl-Meyer Assessment for Upper Extremity; BI: Barthel Index.

For FMA-UE, aDCS (MD = 8; 95% CrI = 2.1, 14), LF-rTMS (MD = 8.3; 95% CrI = 4.3, 13), cTBS (MD = 8.7; 95% CrI = 0.75, 17), and HF-rTMS (MD = 9.6; 95% CrI = 4.5, 15) were all superior to conventional rehabilitation therapy. aDCS (MD = 3.8; 95% CrI = 0.36, 7.4), LF-rTMS (MD = 4.1; 95% CrI = 0.49, 7.6), and HF-rTMS (MD = 5.4; 95% CrI = 0.96, 9.7) were superior to the Sham group. For BI, aDCS was superior to the Sham group (MD = 6.3; 95% CrI = 1.9, 10), conventional rehabilitation therapy (MD = 7.9; 95% CrI = 0.47, 16), and bipolar DCS (MD = 7.8; 95% CrI = 0.21, 15). LF-rTMS was superior to conventional rehabilitation therapy (MD = 6; 95% CrI = 1.7, 11). No other interventions showed statistically significant differences. See Figure 5.

### 3.6 Probability rankings

The GEMTC package in R software was used to create rankograms for the five outcome indicators. Bar charts display the probability ranking of each intervention being the best treatment. Combined with the network results, the final probability rankings are:

FMA-UE: HF-rTMS (29%), cTBS (27%), cDCS (17.5%), aDCS (0.8%), iTBS (0.66%), bipolar DCS (0.65%), LF-rTMS (0.46%), Sham (0), Conventional therapy (0).

BI: aDCS (71.5%), LF-rTMS (20.9%), HF-rTMS (4.4%), iTBS (1.6%), bipolar DCS (1.1%), Conventional therapy (0.17%), Sham (0.03%).

WMFT: aDCS (63.6%), cTBS (13%), HF-rTMS (9.1%), bipolar DCS (8.7%), Conventional therapy (3.2%), LF-rTMS (1.6%), Sham (0.4%).

MAS: iTBS (42%), LF-rTMS (24%), cTBS (16%), bipolar DCS (6.9%), aDCS (3.27%), Conventional therapy (3.26%), HF-rTMS (3%), Sham (0.7%).

ARAT: iTBS (72.6%), aDCS (22.3%), LF-rTMS (2.8%), bipolar DCS (1.8%), Sham (0.4%).

**Figure 6:**
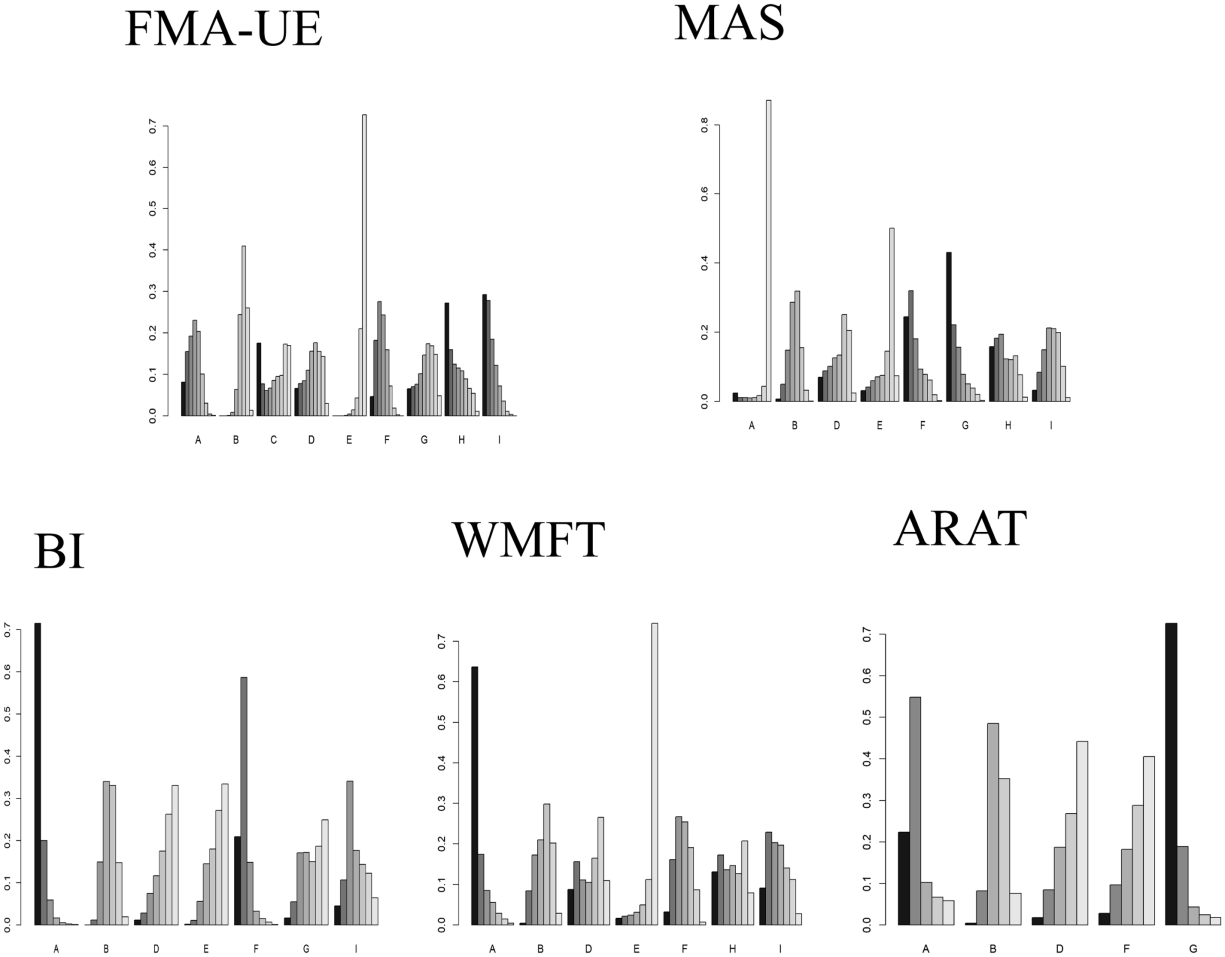
Comparison of the effects of different interventions on different outcome indicators. Different letters on the horizontal axis represent different interventions. The vertical axis represents the probability of each intervention being ranked as the best for the corresponding outcome indicator. The darkest color represents Rank 1. A taller Rank 1 bar indicates a higher probability that the intervention is the best for that outcome indicator. A: anodal transcranial direct current stimulation (aDCS), B: Sham group, C: cathodal transcranial direct current stimulation (cDCS), D: bipolar transcranial direct current stimulation (bipolar DCS), E: Conventional therapy, F: low-frequency repetitive transcranial magnetic stimulation (LF-rTMS), G: intermittent theta burst stimulation (iTBS), H: continuous theta burst stimulation (cTBS), I: high-frequency repetitive transcranial magnetic stimulation (HF-rTMS). FMA-UE: Fugl-Meyer Assessment for Upper Extremity; WMFT: Wolf Motor Function Test; ARAT: Action Research Arm Test; MAS: Modified Ashworth Scale; BI: Barthel Index.

Comparisons of the effects of different interventions on various outcome indicators are shown in Figure 6. The top three interventions for each outcome indicator based on network meta-analysis are shown in Table 6. It is important to note that the rankings for ARAT (6 studies) and MAS (9 studies) are based on limited evidence and their stability may be lower than for outcomes with broader networks. Furthermore, these probability rankings must be interpreted in conjunction with the comparisons in Figure 5. For example, although HF-rTMS ranks first for FMA-UE, its comparisons with other top-ranked interventions like cTBS were not statistically significant. This indicates that while HF-rTMS has the highest probability of being the best, we cannot be statistically confident that it is superior to other interventions.

**Table 6:** Network meta-analysis results of the top three ranked interventions for each outcome measure.

Outcome measure	Top-ranked intervention	Mean difference (MD) vs. placebo (95% CrI)	Second-ranked intervention	Mean difference (MD) vs. placebo (95% CrI)	Third-ranked intervention	Mean difference (MD) vs. placebo (95% CrI)
FMA-UE	HF-rTMS	**(MD = 5.4; 95% CrI = 0.96, 9.7)**	cTBS	(MD = 4.5; 95% CrI = −3.3, 12)	cDCS	(MD = 1.4; 95% CrI = −10, 13)
BI	aDCS	**(MD = 6.3; 95% CrI = 1.9, 10)**	LF-rTMS	(MD = 4.4; 95% CrI = −0.89, 9.7)	HF-rTMS	(MD = 1.1; 95% CrI = −5.6, 7.5)
WMFT	aDCS	(MD = 3.7; 95% CrI = 0.91, 9.9)	cTBS	(MD = 0.57; 95% CrI = −9.9 10)	HF-rTMS	(MD = 0.96; 95% CrI = −6.0, 7.6)
MAS	iTBS	(MD = –0.44; 95% CrI = −1.8, 0.16)	LF-rTMS	(MD = –0.34; 95% CrI = −1.3, 1.3)	cTBS	(MD = –0.16; 95% CrI = −1.5, 1.5)
ARAT	iTBS	(MD = 11; 95% CrI = −2.8, 25)	aDCS	(MD = 5.2; 95% CrI = −7.6, 18)	LF-rTMS	(MD = 1.6; 95% CrI = −10, 14)

Bold text indicates that the 95% credible interval for the comparison does not contain 0.

### 3.7 Publication bias

**Figure 7:**
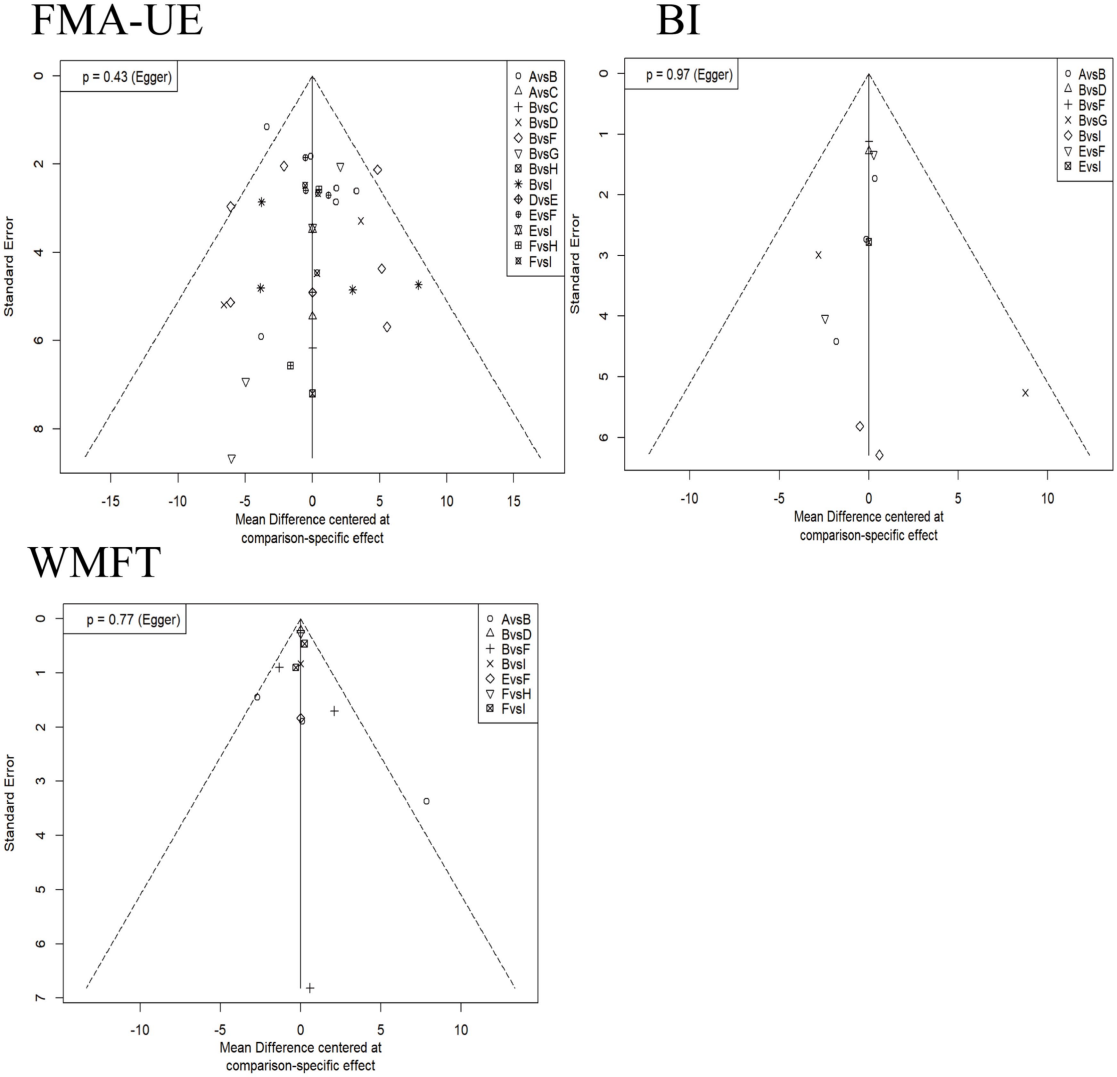
Contour-enhanced funnel plots for each outcome indicator. The horizontal axis represents the mean difference based on specific comparisons, reflecting the effect difference between different interventions. The vertical axis represents the standard error, reflecting the precision of the effect estimate; a smaller standard error indicates a more precise estimate. A: anodal transcranial direct current stimulation (aDCS), B: Sham group, C: cathodal transcranial direct current stimulation (cDCS), D: bipolar transcranial direct current stimulation (bipolar DCS), E: Conventional therapy, F: low-frequency repetitive transcranial magnetic stimulation (LF-rTMS), G: intermittent theta burst stimulation (iTBS), H: continuous theta burst stimulation (cTBS), I: high-frequency repetitive transcranial magnetic stimulation (HF-rTMS). FMA-UE: Fugl-Meyer Assessment for Upper Extremity; WMFT: Wolf Motor Function Test; BI: Barthel Index.

Contour-enhanced funnel plots were drawn, and Egger’s test was performed to assess publication bias. Figure 7 shows the results for different indicators (BI, FMA-UE, WMFT) where the number of studies was >10. The *p*-values for Egger’s test corresponding to each indicator were all greater than 0.05, indicating no statistical significance. This suggests no sufficient evidence of publication bias in these analyses, although some scattered studies indicate possible small-study effects.

### 3.8 Inconsistency check

**Figure 8:**
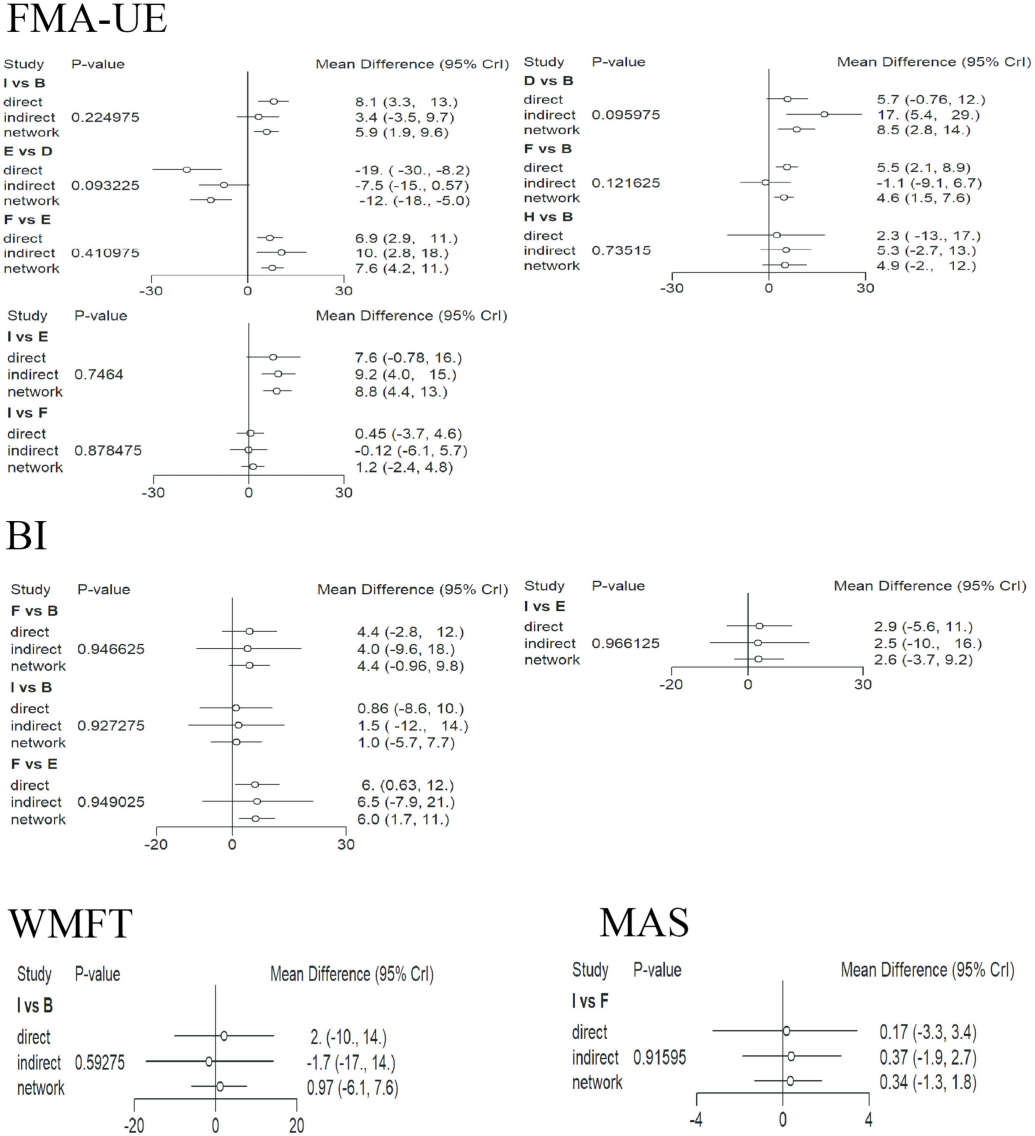
Inconsistency test plots for each indicator. For each comparison, the three points represent the direct, indirect, and network-combined effect estimates, respectively. The lines through the points represent the 95% CrI. A: anodal transcranial direct current stimulation (aDCS), B: Sham group, C: cathodal transcranial direct current stimulation (cDCS), D: bipolar transcranial direct current stimulation (bipolar DCS), E: Conventional therapy, F: low-frequency repetitive transcranial magnetic stimulation (LF-rTMS), G: intermittent theta burst stimulation (iTBS), H: continuous theta burst stimulation (cTBS), I: high-frequency repetitive transcranial magnetic stimulation (HF-rTMS). FMA-UE: Fugl-Meyer Assessment for Upper Extremity; WMFT: Wolf Motor Function Test; MAS: Modified Ashworth Scale; BI: Barthel Index.

The network graphs for FMA-UE, BI, MAS, and WMFT all contained closed loops, so inconsistency tests were performed for these four outcomes. Figure 8 shows the inconsistency test results. The direction of the effect sizes for direct and indirect comparisons was basically consistent for all items, and *p* > 0.05 (no significant inconsistency), indicating the reliability of the comparison results.

## 4 Discussion

This study included 28 randomized controlled trials (RCTs) to systematically evaluate the efficacy of seven non-invasive brain stimulation (NIBS) protocols on upper limb rehabilitation in 1340 stroke patients with varying degrees of upper limb functional impairment. By integrating the five outcome measures mentioned above, it compared and analyzed the effects of these intervention protocols on upper limb motor function, muscle tone, and activities of daily living. Although previous network meta-analyses have compared broad categories of NIBS or focused on specific modalities like rTMS, there is a lack of comprehensive direct and indirect comparisons encompassing contemporary theta burst stimulation paradigms (iTBS and cTBS) alongside traditional rTMS/tDCS. Therefore, this study presents the first Bayesian network meta-analysis that simultaneously integrates and ranks the efficacy of seven specific NIBS techniques across multiple functional domains in post-stroke upper limb rehabilitation. The Bayesian framework provides probability rankings for each intervention, offering a more intuitive interpretation for clinical decision-making regarding the most promising treatment options.

Upper limb motor function is fundamental for performing daily living and occupational activities. Central nervous system damage caused by stroke often leads to upper limb muscle weakness, abnormal muscle tone, and restricted joint range of motion (38). It is noteworthy that upper limb functional recovery typically lags behind lower limb recovery, further exacerbating the decline in patients’ activities of daily living and quality of life (39). This study indicates that HF-rTMS is likely the most effective treatment for improving FMA-UE. Previous research has shown that HF-rTMS can effectively improve upper limb motor function in patients with post-stroke upper limb motor dysfunction. On one hand, it increases cortical excitability by stimulating the ipsilateral primary motor cortex (M1) area, thereby enhancing its excitability and improving upper limb muscle motor function (40). On the other hand, HF-rTMS can promote neuroplasticity, inducing brain functional reorganization, which involves not only the locally stimulated area but also remote cortical and subcortical regions connected functionally (41, 42). Furthermore, HF-rTMS activates motor-related brain areas. Patients receiving this therapy in our study showed significant improvement in Fugl-Meyer Assessment (FMA) scores, indicating enhanced range, quality, and other aspects of upper limb movement. Huang et al. (43), in a network meta-analysis comparing the efficacy of iTBS and HF-rTMS for treating upper limb dysfunction after stroke, found that the probability ranking for improving upper limb dysfunction placed HF-rTMS above iTBS and sham, which is consistent with our results and further confirms our findings. However, the meta-analysis by Shen et al. (44) reported inconsistent results with ours. Our analysis suggests this discrepancy might be related to heterogeneity in their included patient population characteristics and diversity in intervention parameters, among other reasons. Conversely, Khedr et al. (45) applied 3 Hz and 10 Hz rTMS to the affected motor cortex in different patient groups and found no additional advantage of 10 Hz high-frequency stimulation over 3 Hz. This might imply that for high-frequency rTMS (>1 Hz), whether at 3 Hz or 10 Hz, the effect on improving upper limb motor function may not differ.

The minimal clinically important difference (MCID) refers to the smallest change in a target outcome measure that clinicians consider to be clinically meaningful. For the FMA-UE in stroke patients, an improvement of approximately 2–4 points is generally considered clinically significant (46). Our network meta-analysis results show that interventions including HF-rTMS, cTBS, and aDCS are superior to conventional rehabilitation therapy with statistical significance. Although no studies directly comparing the main interventions were included, the MD and CrI from indirect comparisons still suggest that HF-rTMS might be superior to interventions like cTBS and aDCS. Although the point estimate shows the highest MD for HF-rTMS, we cannot confirm its statistical superiority over others like cTBS due to overlapping credible intervals. Nonetheless, their improvement values all exceeded the MCID, suggesting clear clinical value for these interventions. Additionally, the study by Sasaki et al. (47) indicated that in the acute phase, high-frequency stimulation of the affected hemisphere is more effective than low-frequency stimulation of the unaffected hemisphere. Most of the HF-rTMS studies we included involved high-frequency stimulation of the affected hemisphere.

During post-stroke neural remodeling, the cerebral cortex may experience abnormal increases in corticospinal tract excitability due to disinhibition effects, leading to hyperactive stretch reflexes and pathological increases in flexor muscle tone in the affected upper limb (48, 49). Based on limited evidence, intermittent theta burst stimulation (iTBS) appears most beneficial for improving muscle tone regulation (MAS) and fine motor ability (ARAT), significantly improving upper limb spasticity and promoting functional recovery, suggesting its potential advantage in post-stroke upper limb rehabilitation. Although the mechanism of iTBS is not fully understood, existing evidence suggests it may regulate presynaptic calcium channel activity, activate downstream signaling pathways, induce upregulation of synaptic plasticity-related genes and proteins, ultimately leading to adaptive remodeling of synaptic structure and function (50, 51). It is important to note that iTBS and continuous theta burst stimulation (cTBS) have bidirectional regulatory effects on cortical synaptic transmission efficacy: iTBS can rapidly enhance excitability (52), while cTBS tends to induce long-term depression (53). Further mechanistic analysis suggests that the long-term potentiation (LTP) phenomenon mediated by iTBS might promote functional reorganization of the cortical-thalamic-basal ganglia circuit, enhancing the synergistic effect between affected limb motor control and task-specific training (54), which could be an important neural basis for its significant improvement in ARAT scores. Supplementary research by Kim et al. (55) suggested that iTBS might modulate GABAergic interneuron activity, increasing the conduction efficiency of cortical to spinal inhibitory pathways, thereby reducing gamma motor neuron excitability and alleviating spasticity. The study by Ackerley et al. (56) indicated that iTBS with parameters (intra-burst frequency 50 Hz, inter-burst frequency 5 Hz, 90% AMT, total pulses 600) applied to the ipsilateral M1 area significantly improved symptoms like hand stiffness and numbness. Furthermore, Zhang et al. (57) conducted a meta-analysis on the short-term and long-term effects of rTMS on upper limb motor function after stroke. Their analysis concluded that for improving upper limb function and promoting normalization, iTBS is superior to cTBS, which aligns with our findings. However, Chen et al. (58) conducted a meta-analysis on iTBS for post-stroke motor dysfunction, and their results showed that iTBS had significant statistical meaning for improving fine motor ability (ARAT) but not for improving muscle tone regulation (MAS). This discrepancy might be due to their study’s focus on all patients with post-stroke motor dysfunction, including those with both upper and lower limb impairments, leading to study differences. Additionally, Chen et al. (59) considered the MCID for MAS to be 0.48 for a medium clinical effect and 0.76 for a large clinical effect. Amano et al. (60), using the Japanese version of ARAT, suggested the MCID for ARAT is approximately 5.7 points. Our network meta-analysis results show that the MD values for many interventions are greater than the MCID values. However, since these direct or indirect comparisons lack statistical significance, these results need to be interpreted cautiously.

Anodal transcranial direct current stimulation (aDCS) may rank first for both activities of daily living (BI) and motor task performance (WMFT). This effect might involve dual neuroregulatory pathways: (1) Primary motor cortex (M1) excitability modulation: Anodal stimulation may induce depolarization of neuronal membrane potentials, enhancing the electrical activity level of neurons in the M1 area, thereby improving synaptic plasticity in the corticospinal pathway and optimizing motor signal transmission efficiency (40). (2) Multi-brain network synergistic remodeling: The neural remodeling effects mediated by aDCS might involve the reorganization of dynamic connectivity between the default mode network and the motor network, potentially by reducing interference from non-task-related brain areas, thereby improving the learning speed and execution accuracy of task-oriented movements (61). These mechanisms can manifest in the Wolf Motor Function Test (WMFT) as reduced task completion time and simultaneous improvement in the Functional Ability Scale score. Our findings are consistent with the meta-analysis by Yu et al. (62), who conducted a systematic review on the efficacy of aDCS for upper limb function after ischemic stroke. Their results indicated that aDCS positively impacts both BI and WMFT, suggesting it is an effective and relatively safe intervention. The meta-analysis by Tang et al. (63) showed that aDCS is most effective in subacute stroke patients, with C3/C4 being the most effective stimulation target, an optimal stimulation duration of 20–30 min, and fewer than 30 sessions. This further corroborates our findings. Additionally, Hsieh et al. (64) studied the MCID for the Barthel Index (BI) in assessing stroke patients’ daily activities, finding an MCID of 1.85 for the BI. Lin et al. (65) reported an MCID of 0.37 for WMFT. According to our results, for improving BI levels, both aDCS and LF-rTMS interventions are superior to conventional rehabilitation therapy with statistical significance. Based on the MD and CrI from direct or indirect comparisons, aDCS might be superior to LF-rTMS, and their improvement values exceed the MCID, indicating clear clinical value for these interventions. Regarding WMFT, the network meta-analysis results show that the MD values for many interventions are greater than the MCID value. However, since these direct or indirect comparisons lack statistical significance, these results also need to be interpreted cautiously.

### 4.1 Strengths and limitations

To our knowledge, this is the first network meta-analysis (NMA) to compare and rank the efficacy of seven intervention protocols, including a comprehensive direct and indirect comparison of (iTBS and cTBS) with traditional rTMS/tDCS, for patients with upper limb motor dysfunction after stroke. Furthermore, utilizing NMA can enhance evidence-based clinical decision-making, as NMA combines direct and indirect comparisons from trials. This methodology provides ranking results indicating the relative effectiveness of each intervention type, aiding informed decision-making. Additionally, focusing specifically on studies enhancing motor function is considered a valuable addition to the NMA in terms of statistical completeness. Overall, the primary objective of this NMA is to provide better rehabilitation strategies for patients and stronger decision-making support for clinicians.

Nonetheless, our study does have several limitations.

1. The number of studies investigating the effects of different treatments varied significantly, with particularly limited evidence for fine motor dysfunction (ARAT) and spasticity (MAS) (ARAT *n* = 6, MAS *n* = 9). In networks with small sample sizes, the precision and stability of estimates are affected. Therefore, conclusions regarding the relative efficacy of interventions for fine motor dysfunction (ARAT) and spasticity (MAS) should be considered preliminary and require validation through larger studies.
2. A relatively high proportion of small-sample RCTs originated from China, and there was a lack of long-term follow-up data. While these studies enriched the network quantitatively, their methodological rigor is generally inferior to international multicenter large-sample studies, which might lead to an overestimation of the overall effect size.
3. There is potential for publication bias and language bias. Although we systematically searched Chinese and English databases, we may have missed unpublished negative results or studies in other languages.
4. Novel techniques such as transcranial alternating current stimulation and transcranial random noise stimulation were included in the search but did not enter the final analysis due to the inclusion and exclusion criteria.
5. Although sham stimulation in most studies was confirmed to effectively maintain participant blinding, there remains a potential risk to blinding integrity if participants had prior experience with stimulation therapy, as they might discern group assignment based on perceived differences in duration or intensity.
6. For rTMS, while we analyzed low-frequency and high-frequency stimulation as separate interventions, each node itself still encompasses different parameter spectra. Inconsistencies in stimulation intensity, and the lack of uniformity in using resting threshold or active motor threshold, make it impossible to determine whether the net energy actually delivered to the cerebral cortex is comparable. Simultaneously, the total number of pulses, session duration, and treatment cycle also lack unified standards. This “dose” uncertainty makes it difficult for us to draw definitive conclusions about the optimal treatment protocol. Similar issues exist in the tDCS domain. Electrode montage is a key factor determining current flow and thus the brain areas affected. Our comprehensive analysis included studies with the anode placed over the M1 area or premotor cortex, and the cathode over the contralateral shoulder or supraorbital region. Furthermore, the efficacy of tDCS is cumulative and dose-dependent; differences in the total number of sessions and treatment cycles further complicate the analysis. Although this report provides a valuable macro-perspective for the field of neurology, readers should interpret the efficacy rankings cautiously. Future research could shift toward meta-analyses investigating the effects of non-invasive brain stimulation on upper limb motor function in patients at different stroke stages and the impact of optimal parameters to further validate efficacy.

## 5 Conclusion

This study systematically evaluated the multidimensional efficacy of non-invasive brain stimulation on upper limb functional rehabilitation after stroke. The results demonstrate significant heterogeneity in the efficacy of different interventions across specific functional domains:

High-frequency repetitive transcranial magnetic stimulation (HF-rTMS) is most likely to be the most effective intervention for restoring motor function (FMA-UE); a mechanism potentially related to the potent activation of the corticospinal tract and enhanced synaptic plasticity by high-frequency stimulation.

Anodal transcranial direct current stimulation (aDCS) may rank first for both activities of daily living (BI) and motor task performance (WMFT); suggesting it significantly enhances functional independence by increasing excitability in the primary motor cortex (M1) and optimizing dynamic connectivity within motor networks.

Intermittent theta burst stimulation (iTBS) appears beneficial for improving muscle tone regulation (MAS) and fine motor ability (ARAT), potentially stemming from its specific modulation of cortical inhibitory interneurons.

However, the results for ARAT and MAS, based on a limited number of studies, require cautious interpretation due to constrained evidence.

## Data Availability

The original contributions presented in the study are included in the article/Supplementary material, further inquiries can be directed to the corresponding authors.
