# Supplementary material for: Efficacy comparison of seven non-invasive brain stimulation techniques for upper limb motor dysfunction after stroke: a Bayesian network meta-analysis and systematic review
Source: Front Neurol. 2025 Dec 19;16:1693537. doi: 10.3389/fneur.2025.1693537 (PMC12801347; doi:10.3389/fneur.2025.1693537)
Supplement: Supplementary file 1 [file Data_Sheet_1.PDF]

Supplementary Table 1

Author (Year)	Journal	Patient Source / Recruitment Details
X Han2023(15)	Chinese Journal of Rehabilitation	96 stroke patients enrolled from the Department of Rehabilitation Medicine, Capital Medical University Electric Power Teaching Hospital (June 2018 - March 2022).
C,J Zheng2019(19)	Chinese Journal of Rehabilitation	281 stroke patients with upper limb motor dysfunction from Hubei Provincial Integrated Traditional Chinese and Western Medicine Rehabilitation Center and Neurology Department (March 2017 - December 2018).
F,B Sun2023(16)	Chinese Journal of Rehabilitation Theory and Practice	60 stroke patients from the Department of Rehabilitation Medicine, Zhejiang Provincial People's Hospital (June 2021 - December 2022).
Y Yin2015(18)	Chinese Journal of Rehabilitation Theory and Practice	Stroke patients recruited from the Rehabilitation Department of Hebei Provincial People's Hospital (April 2012 - January 2013).

B,J Li2016(29)	Chinese Journal of Rehabilitation Theory and Practice	60 patients with upper limb hemiplegia after stroke from the Neurorehabilitation Center, Beijing Bo'ai Hospital, China Rehabilitation Research Center (December 2014 - December 2015).
Y Liu2018(31)	Chinese Journal of Rehabilitation Theory and Practice	23 patients with post-stroke upper limb spastic hemiplegia receiving rehabilitation in the Neurology Department and Traditional Chinese Medicine Physiotherapy Department of Fuzhou General Hospital of the Chinese PLA (September 2015 - December 2017).
X,M Meng2016(32)a	Chinese Journal of Rehabilitation Theory and Practice	51 patients with cerebral infarction from the Rehabilitation Medicine Department, Huangdao Branch, Affiliated Hospital of Qingdao University (September 2014 - July 2015).
C,L Xiao2019(35)	Chinese Journal of Rehabilitation Theory and Practice	60 stroke inpatients from the Rehabilitation Medicine Department, the Second Affiliated Hospital of Guangzhou Medical University (June 2016 - September 2017).

Z Zhou2020(36)	Chinese Journal of Rehabilitation Theory and Practice	60 stroke inpatients from Shanghai First Rehabilitation Hospital (August 2018 - July 2019).

Supplementary Table 2

Author (year)	Sham stimulation method
PiresR.2023(10)a	The same montage and stimulation parameters were employed for sham stimulation, however, current was only applied for 30 s to induce the slight tingling sensation that some subjects report experiencing during tDCS stimulation. Furthermore, for both real and sham stimulation current intensity was gradually increased (at the beginning of the session) during 10 s and decreased (at the end of the session) also during 10 s to diminish its perception.
DiLazzaro,V.2014(12)	The same montage and stimulation parameters were employed for sham stimulation, however, current was only applied for 30 s to induce the slight tingling sensation that some subjects report experiencing during tDCS stimulation. Furthermore, for both real and sham stimulation current intensity was gradually increased (at the beginning of the session) during 10 s and decreased (at the end of the session) also during 10 s to diminish its perception.
Koh,C.L.2017(13)	The device parameter settings for the control intervention were identical to those of the tDCS-SM group. Sham bilateral tDCS was administered by delivering direct current only during the

	first 30 seconds; studies have shown that this duration effectively maintains participant blinding to the intervention group assignment without altering cortical excitability.
Lindenberg,R.2010(14)	The same montage and stimulation parameters were employed for sham stimulation, however, current was only applied for 30 s to induce the slight tingling sensation that some subjects report experiencing during tDCS stimulation. Furthermore, for both real and sham stimulation current intensity was gradually increased (at the beginning of the session) during 10 s and decreased (at the end of the session) also during 10 s to diminish its perception.
X Han2023(15)	The same montage and stimulation parameters were employed for sham stimulation, however, current was only applied for 30 s to induce the slight tingling sensation that some subjects report experiencing during tDCS stimulation. Furthermore, for both real and sham stimulation current intensity was gradually increased (at the beginning of the session) during 10 s and decreased (at the end of the session) also during 10 s to diminish its perception.
F,B Sun2023(16)	The same montage and stimulation parameters were employed for sham stimulation, however, current was only applied for 30 s to induce the slight tingling sensation that some subjects report experiencing during tDCS stimulation. Furthermore, for both real and sham stimulation current intensity was gradually increased (at the beginning of the session) during 10 s and decreased (at the end of the session) also during 10 s to diminish its perception.

Q,J Wang2018(17)	The same montage and stimulation parameters were employed for sham stimulation, however, current was only applied for 30 s to induce the slight tingling sensation that some subjects report experiencing during tDCS stimulation. Furthermore, for both real and sham stimulation current intensity was gradually increased (at the beginning of the session) during 10 s and decreased (at the end of the session) also during 10 s to diminish its perception.
Y Yin2015(18)	The same montage and stimulation parameters were employed for sham stimulation, however, current was only applied for 30 s to induce the slight tingling sensation that some subjects report experiencing during tDCS stimulation. Furthermore, for both real and sham stimulation current intensity was gradually increased (at the beginning of the session) during 10 s and decreased (at the end of the session) also during 10 s to diminish its perception.
C,J Zheng2019(19)	The same montage and stimulation parameters were employed for sham stimulation, however, current was only applied for 30 s to induce the slight tingling sensation that some subjects report experiencing during tDCS stimulation. Furthermore, for both real and sham stimulation current intensity was gradually increased (at the beginning of the session) during 10 s and decreased (at the end of the session) also during 10 s to diminish its perception.
Rose,D.K.2014(20)	Sham-rTMS was delivered using a placebo coil that looks like

	and imitates the sound of a real coil.
Chen,Y.2021(21)	For sham stimulation, the stimulation coil was rotated 90° so that the minimal current flow was induced in the brain, and it was still centered on the same scalp position with the same parameter as the cerebellar iTBS group.
Chen,Y.H.2021(22)	Sham stimulation was administered at the same site with identical flip coil, resulting in a 78% output elicited by non-flip side, at a lower intensity (60% AMT) equivalent to 46.8% AMT . The sham stimulation with intensity lower than 70% AMT has no effect on MEPs, as demonstrated by a previous study , but produces indistinguishable sensation and sound compared to real stimulation.
Chen,Y.J.2019(23)	Sham stimulation was administered on the same site with the coil flipped over at a lower intensity (60% AMT), which results in a lower output equivalent to that of the normal site at approximately 46.8% AMT, because the output of the flip side is about 78% of the normal side.
Kuzu,Ö2021(25)a	Real TMS applications were performed with eight-shaped 70 mm coil and sham TMS applications were performed with sham coil which has a similar appearance with the active coil.
Li,J.2016(26)a	We used a false coil (offered by YRD Company) for the sham stimulation, which delivered negligible magnetic output with audible click-on discharge. As the patients had not previously undergone TMS treatment, they were naturally blinded as to sham or actual treatment.

Senió́w,J.2012(28)	Immediately before a 45-minute physiotherapy session, patients from E group were subjected to 30 minutes of rTMS applied to the hand area of M1 in the unaffected hemisphere to suppress its excitability. The C group received placebo stimulation.
X,M Meng2016(32)a	The sham stimulation group received placebo coil stimulation on the affected hemisphere, with a stimulation frequency of 10 Hz and an intensity of 80% MT, delivered using the Yiruide CYY-I magnetic stimulator (maximum output intensity: 3 T; coil diameter: 12.5 cm).
X,W Tang2018(33)	For sham stimulation, the stimulation coil was rotated 90° so that the minimal current flow was induced in the brain, and it was still centered on the same scalp position with the same parameter as the cerebellar iTBS group.
Y,Q Wang2020(34)	The sham stimulation group received treatment with parameters identical to the conventional group, but with the coil placed perpendicular to the patient's scalp.
C,L Xiao2019(35)	Sham stimulation was administered with patients in the supine position. The stimulation site, frequency, duration, and treatment course were identical to the observation group. During treatment, the stimulation coil was rotated 90 degrees to be perpendicular to the scalp surface.
Z Zhou2020(36)	Patients received rTMS sham stimulation in the supine position. The stimulation site, frequency, duration, and treatment course were identical to the observation group. During treatment, the stimulation coil was rotated 90 degrees to be perpendicular to the scalp surface.
